# Improving the efficiency of trials using innovative pilot designs: the next phase in the conduct and reporting of pilot and feasibility studies

**DOI:** 10.1186/s40814-017-0159-2

**Published:** 2017-07-06

**Authors:** Lehana Thabane, Gillian Lancaster

**Affiliations:** 1 Department of Health Research Methods, Evidence, and Impact, McMaster University, Hamilton, ON Canada; 2 Professor Gillian Lancaster, Institute of Primary Care and Health Sciences, Keele University, Keele, UK

## Abstract

With continuously increasing costs of conducting trials, use of innovative approaches—such as pragmatic trials, registry-based randomised trials, adaptive trials, personalised medicine trials, platform trials, and basket trials—to the design and conduct of clinical trials has been advocated as one of the most promising solutions. In this editorial, we propose that the next wave of feasibility or pilot studies should focus on assessing the feasibility of trials using these designs, which we see as an imperative in order to unleash their potential to reduce trial costs and accelerate the drug development process and the search for best treatments, so that the right treatments can be delivered as soon as possible to the right patients.

In this editorial, we welcome Professor Lehana Thabane as new co-Editor-in-Chief to work alongside Professor Gillian Lancaster. The journal has seen substantial growth over the past 18 months and with it the need for a second Editor-in-Chief. With all articles published in the journal indexed in PubMed, we look to the future and consider the next phase in the conduct and reporting of pilot and feasibility studies; we think about how innovative trial designs are contributing to improving trial efficiency.

*Pilot and Feasibility Studies* was launched in January 2015 *to provide a dedicated place for the reporting of feasibility and pilot studies, and discussion of methodological issues around the planning, of future large-scale definitive trials and observational studies* [1]. Led by the Working Group of the CONSORT Extension for Pilot and Feasibility Trials, the journal was established as part of the collective effort to change the practice of reporting of pilot and feasibility studies [2]. These efforts have also led to the publication of a framework which clarifies the similarities and differences between a feasibility study and a pilot study. The former asks whether something can be done, if we should we proceed with it, and if so, how? In contrast, the latter asks the same questions, but it also has the specific design feature that a pilot study is a forerunner of a future study (or part of a future study) which will be conducted on a larger scale [3].

Then followed the publication of the CONSORT extension to pilot and feasibility trials, aimed at enhancing the transparency and completeness of reporting of pilot and feasibility randomised clinical trials, which are designed to inform the planning of future definitive trials [4, 5]. The methods and processes used to develop the CONSORT guideline have been described elsewhere [6]. Since its inception, *Pilot and Feasibility Studies* has received numerous submissions of pilot work, leading to over 140 publications—with 89 study protocols and 44 research reports of pilot and feasibility studies as the most frequent types of publication (Table 1). Clearly, the publication of pilot work in the journal is gaining momentum. This is also a sign of the growing recognition of the importance of reporting the design and results of pilot and feasibility studies.

**Table 1:** Type of publications in *Pilot and Feasibility Studies* since 2015

Article type	2015	2016	2017 (Jan–June)	Total
Study protocol	25	45	18	89
Research results	14	20	10	44
Editorial	2	0	0	2
Review	1	1	1	3
Methodology	1	3	1	5
Commentary	0	1	0	1
Letter	0	1	0	1
Update	0	1	0	1

Data from journal website on 13 June 2017

With rapidly rising costs of conducting clinical trials and limited funding [7], there has been some intense debate among stakeholders—funders, sponsors, clinical trialists, consumers and providers—on how to reduce trial costs. The use of innovative approaches to the design and conduct of clinical trials has been advocated as one of the most promising solutions. Examples of these approaches include pragmatic trials [8], observational trials [9], registry-based randomised trials [10], cluster randomised trials [11], adaptive trials [12], trials using Bayesian methods [13], small sample, biologically based trials (e.g. personalised medicine trials) [14], N-of-1 trials [15], platform trials [16], basket trials [17, 18] and umbrella trials [18]. In general, these innovations seem intuitively appealing. For example, registry-based trials—which use registries as the platform for data collection, randomization, and follow-up [10]—have even been described as *the next disruptive technology in clinical research* [19]; the platform trial, whose goal is to find the best treatment or management for a disease condition by simultaneously studying multiple treatments, has been characterised as *a vision for the future* [16].

Compared to standard clinical trial methodologies, these innovations offer many advantages. For example, because of their flexibility and efficiency, adaptive trial designs [12] offer the potential to cut drug development costs by allowing researchers (i) the opportunity to correct or update incorrect assumptions that were made at the start of the trial; (ii) to select the most promising treatment options as early as possible; (iii) to use emerging information that is external to the trial, in order to adapt the design; and (iv) the opportunity to react sooner to surprising results—for efficacy, safety or futility. These adaptations can shorten the development time and consequently speed up the development process, and lower costs. Another example is the use of basket trials in oncology [17, 18], which have the advantage of utilising novel designs that match patients with different cancer types (“baskets”) that have a rare genetic mutation, regardless of tumour histology, to a drug that is expected to work through the mutational pathway.

However, there remain several uncertainties about the feasibility of some of these innovations in many clinical or health care settings. Therefore, assessing the feasibility and the prevailing uncertainties of the applications of these state-of-the-art technologies and strategies to clinical trials should be a top priority. These include feasibility of being able to recruit trial participants; implementation of intervention elements; assessment of outcomes; and evaluation of the acceptability and ethics of the designs; among others. As we look into the future, we can expect the next wave of pilot and feasibility studies to focus on these uncertainties, in order that the promise of reducing costs by using innovative trial designs can be realised. In fact, we have already seen a number of examples of pilot trials which have assessed the feasibility of different aspects of these designs. These have included the BRAVE pilot trial [20] which used a pragmatic design to assess the feasibility of a behavioural activation group therapy in reducing depressive symptoms and improving quality of life in patients with depression; a study aiming to determine the feasibility of using patient/disease registries to recruit subjects for clinical trials [21]; a pilot study using Bayesian methods in N-of-1 trials to assess the effect of Amitriptyline to relieve pain in juvenile idiopathic arthritis [15]; to mention only a few.

The application of innovative trial designs has the potential to reduce trial costs and accelerate the drug development process and the search for best treatments, so that the right treatments can be delivered as soon as possible to the right patients. But for these designs to have a substantial impact on how we develop new treatment strategies, our approaches have to start with assessing their feasibility, and adopting the right objectives and outcomes. Success in assessing feasibility will add to our evolving scientific knowledge base, and to our understanding of the ecology of patient care and the biology of human disease; and it will, hopefully, allow us to better design these trials to enhance the tailoring of more treatments to more patients in the future. As we move into the next phase of the new era in the conduct and reporting of pilot and feasibility studies, we look forward to continuing to work with the editorial team, to place *Pilot and Feasibility Studies* in the forefront of research innovation, and allow it to act as a useful forum for the dissemination and discussion of pilot or feasibility studies, particularly those that use innovative trial designs. We should mention that the journal is also a forum for publishing qualitative work and process evaluation alongside trial design in any pilot work [22]; and debates on topical issues related to the design, conduct and reporting of pilot and feasibility studies [23].
